# Inertial range of magnetorotational turbulence

**DOI:** 10.1126/sciadv.adp4965

**Published:** 2024-08-28

**Authors:** Yohei Kawazura, Shigeo S. Kimura

**Affiliations:** ^1^ Frontier Research Institute for Interdisciplinary Sciences, Tohoku University, 6-3 Aoba, Aramaki, Sendai 980-8578, Japan.; ^2^ Department of Geophysics, Graduate School of Science, Tohoku University, 6-3 Aoba, Aramaki, Sendai 980-8578, Japan.; ^3^ School of Data Science and Management, Utsunomiya University, 350 Minemachi, Utsunomiya, Tochigi 321-8505 Japan.; ^4^ Astronomical Institute, Tohoku University, 6-3 Aoba, Aramaki, Sendai 980-8578, Japan.

## Abstract

Accretion disks around compact stars are formed due to turbulence driven by magnetorotational instability. Despite over 30 years of numerous computational studies on magnetorotational turbulence, the properties of fluctuations in the inertial range—where cross-scale energy transfer dominates over energy injection—have remained elusive, primarily due to insufficient numerical resolution. Here, we report the highest-resolution simulation of magnetorotational turbulence ever conducted. Our simulations reveal a constant cross-scale energy flux, a hallmark of the inertial range. We found that as the cascade proceeds to smaller scales in the inertial range, the kinetic and magnetic energies tend toward equipartitioning with the same spectral slope, and slow magnetosonic fluctuations dominate over Alfvénic fluctuations, having twice the energy. These findings align remarkably with the theoretical expectations from the reduced magnetohydrodynamic model, which assumes a near-azimuthal mean magnetic field. Our results provide important implications for interpreting the radio observations by the Event Horizon Telescope.

## Teaser

Supercomputer simulation reveals that accretion disks are Alfvénic turbulence with predominantly slow magnetosonic fluctuations.

## INTRODUCTION

Accretion disks around compact stars, such as black holes, neutron stars, and young stars, represent one of the most intriguing phenomena in astrophysics. For matter to accrete onto a compact star, the angular momentum of the matter must be transported outward. It is widely believed that this angular momentum transport is achieved through turbulence driven by magnetorotational instability (MRI) (*1*). Magnetorotational turbulence is such a rich process that it is not only crucial for angular momentum transport but also plays a critical role in the heating and acceleration of particles through dissipation of electromagnetic fluctuations (*2*–*6*), and the energized particles are thought to be responsible for the observed emissions from these systems. To understand particle energization, it is essential to elucidate the properties of turbulence in the inertial range (*7*–*10*), which bridges the energy injection scales and the dissipation scales. In the inertial range, both energy injection and dissipation are subdominant compared to the cross-scale energy transfer by nonlinear effects.

While numerous studies numerically explored the turbulence driven by MRI over 30 years (*11*–*16*), the properties of fluctuations in the inertial range remain unknown. For example, there is a clear discrepancy between the energy spectra in magnetorotational turbulence and those in the theoretical expectations of magnetohydrodynamic (MHD) turbulence (*17*, *18*). The discrepancy is attributed to the insufficient numerical resolution (*14*), although the statistical analysis of intermittent small-scale structures indicates that the spectra would eventually be consistent with those of MHD turbulence at a sufficiently high numerical resolution (*19*). In addition to the energy spectra, the energy partitioning of the MHD modes in the magnetorotational turbulence has not been investigated yet, whereas the partition has been numerically investigated in the MHD turbulence with artificial forcing (*20*–*22*). The energy partition of the MHD modes is important for understanding the ion-to-electron heating ratio (*10*), which is crucial for interpreting the radio observations by the Event Horizon Telescope. The energy partition of the MHD modes also affects the particle acceleration efficiency in accretion flows (*23*, *24*), which has a strong influence on the high-energy neutrino signals from nearby active galactic nuclei (*25*–*27*).

Recently, these two mysteries in magnetorotational turbulence, namely, the energy spectra and the partition of MHD modes, were resolved using the reduced MHD model (*28*), which assumes the presence of a near-azimuthal mean magnetic field. More specifically, both the kinetic and magnetic energy spectra approach *k*^−3/2^ with the same amplitude as the cascade proceeds where *k* is the wave number, and the energy flux of the slow magnetosonic fluctuations is almost double that of the Alfvénic fluctuations. This study aims to resolve the inertial range of magnetorotational turbulence by leveraging the power of the world’s fastest supercomputer and to examine whether the predictions made by the reduced MHD are valid or not.

## RESULTS

Here, we present the direct numerical simulation of magnetorotational turbulence with the highest resolution in history. The simulation was performed on the Fugaku supercomputer (the world’s fastest machine until May 2022), using approximately 128 million central processing unit (CPU) hours. We solved the incompressible MHD equations using a pseudospectral method in a local shearing box with *N_x_* × *N_y_* × *N_z_* = 8192 × 8192 × 4096 grid points, where *x*, *y*, and *z* denote radial, azimuthal, and vertical directions, respectively. The initial magnetic field was set to be vertically uniform with small-amplitude perturbations. The size of the box was set to *L_x_* × *L_y_* × *L_z_* = 4λ × 8λ × 2λ, where λ = 2π*v*_A_/Ω approximately equals the wavelength of the fastest-growing modes of MRI, *v*_A_ is the Alfvén speed given by the initial magnetic field, and Ω is the angular velocity of the accretion disk. We note that the size of our simulation box is relatively smaller than those used in other simulations. This configuration was chosen because our objective is to investigate the inertial range, which is expected to appear at scales smaller than λ. A limitation of using a smaller box size is the inability to estimate the saturation amplitude of fluctuations and angular momentum transport, α. Therefore, we do not discuss these issues in this paper. To narrow the dissipation range, the cascade was terminated by fourth-order hyperviscosity and hyperresistivity. The viscous and resistive coefficients are set to the same value (i.e., magnetic Prandtl number Pm is unity). Further details of the numerical setup can be found in Methods.

### Morphology of fluctuations

**Fig. 1.**
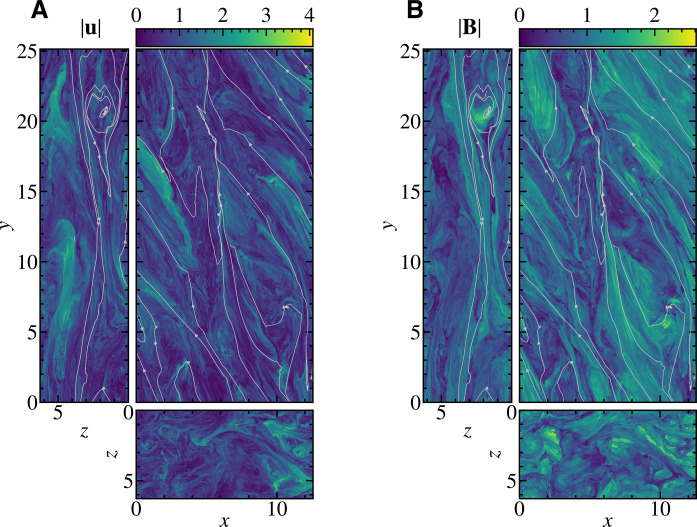
Morphology of finely resolved spatial structures of magnetorotational turbulence. Spatial distributions of (**A**) flow and (**B**) magnetic field intensity on *x* = 0, *y* = 0, and *z* = 0 planes, where *x*, *y*, and *z* denote radial, azimuthal, and vertical directions, respectively. The white lines are typical magnetic field lines.

**Fig. 2.**
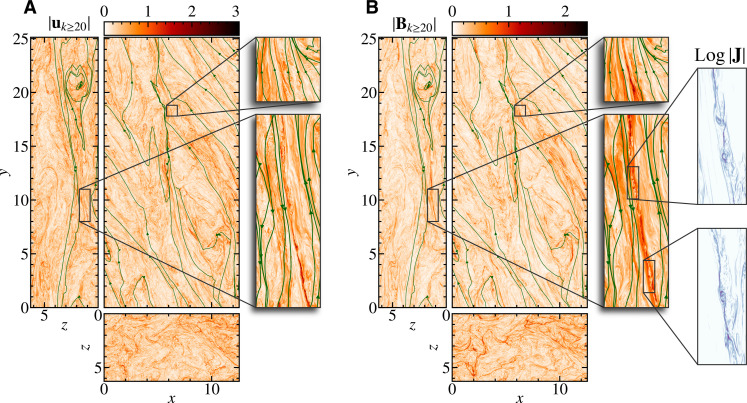
Small-scale structure of fluctuations. The same snapshot as Fig. 1, but high-pass filtered at ∣**k**∣ ≥ 20 (as shown below, the fluctuations with ∣**k**∣ ≥ 20 are in the inertial range). (**A**) and (**B**) correspond to the flow and magnetic field intensity distributions, respectively, The bluish pseudo color in (B) indicates the magnitude of unfiltered electric current density **J** in a logarithmic scale. The green lines are typical unfiltered magnetic field lines.

Figure 1 (A and B) shows the snapshot of the norm of the flow field **u** and the magnetic field **B** together with typical magnetic field lines, in the planes *x* = 0, *y* = 0, and *z* = 0. Each field is normalized by its root mean square value. The overall structures of both fields are azimuthally elongated due to the Keplerian shear flow. Upon closer inspection, the magnetic field has broad structures, while the flow field is concentrated in relatively smaller patches. The reason for this difference is that the large-scale azimuthal magnetic field *B_y_* is preferentially amplified by the Ω effect (*15*), which is evident from the shape of magnetic field lines on the *x* = 0 and *z* = 0 planes (see also fig. S1 for the snapshot of all components of **u** and **B** showing that only *B_y_* has intense large-scale structures). One also notices that the magnetic field lines are predominantly azimuthal but have a finite radial component with anticorrelation between *B_x_* and *B_y_* [consistent with the hybrid particle-in-cell simulation of the shearing box magnetorotational turbulence (*29*); see also fig. S1 (A and B)]. Figure 2 (A and B) shows the same snapshot of **u** and **B** but high-pass filtered by removing the fluctuation with a wave number smaller than 20. As we will show later, the fluctuations with the wave number greater than 10 are arguably in the inertial range. The filtered **u** and **B** exhibit similar morphology, unlike their unfiltered counterparts, suggesting that the cascade tends to be Alfvénic as it moves toward smaller scales. It is also observed that the spatial structures of the filtered fields are elongated along the magnetic field lines. The magnified regions in Fig 2 (A and B) clearly visualize this elongation. Thus, the large-scale magnetic field effectively acts like a mean field for the fluctuations in the inertial range. One also finds that the azimuthal polarity of the mean magnetic field is not uniform, which is evident from the typical magnetic field lines plotted in Figs. 1 and 2. This is because MRI creates both positive and negative *B_x_*, which turn into positive and negative *B_y_* via shear flow. Therefore, the emergence of the current sheet and magnetic reconnection of a mean field are naturally expected to occur in the simulation, and we find the chain of multiple plasmoids in the magnified region on the *x* = 0 plane in Fig. 2B. Nevertheless, these plasmoids do not fill a substantial portion of the simulation domain but appear only in limited regions.

### Spectral properties

**Fig. 3.**
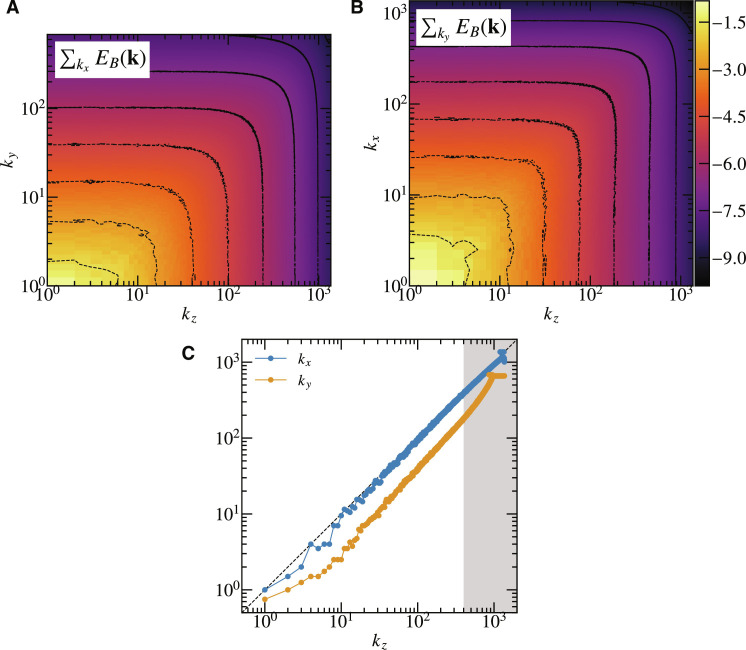
Wave number anisotropy of magnetic energy spectrum. Two dimensional contour of magnetic energy spectrum on (**A**) (*k_z_*, *k_y_*) plane integrated over *k_x_* and on (**B**) (*k_z_*, *k_x_*) plane integrated over *k_y_*. The snapshot was taken immediately after remapping from the shearing coordinate to the laboratory coordinate so that the radial wave number *k_x_* in the both coordinate coincides. The colorbar applies to both (A) and (B). (**C**) *k_z_* intercept versus *k_y_* and *k_x_* intercepts of contour lines in (A) and (B). The gray shaded area indicates to the dissipation range.

To explore the properties of small-scale fluctuations, we analyzed the energy spectra of **u** and **B**. Figure 3 (A and B) presents the two-dimensional spectrum of magnetic energy. Figure 3A shows the spectrum as a function of *k_z_* and *k_y_*, with the *k_x_* direction integrated out, while Fig. 3B displays the spectrum as a function of *k_z_* and *k_x_*, integrating out the *k_y_* direction. The snapshot was taken immediately after remapping from the shearing coordinate to the laboratory coordinate, so that the radial wave number *k_x_* in both coordinates coincides (see Methods for details about the periodic remapping and the time dependency of *k_x_*). The results indicate anisotropy, specifically *k_x_* ≃ *k_z_* > *k_y_*. This aligns with the observation seen in Figs. 1 and 2 that the fluctuations are elongated along the shear and the mean magnetic field on the *x-y* and *y-z* planes, while the structures are nearly isotropic on the *x-z* plane. However, Fig. 3C, which displays *k_x_* and *k_z_* as functions of *k_z_* by flattening Fig. 3 (A and B), shows that the anisotropy is scale independent, unlike the scale-dependent anisotropy commonly seen in other simulations of MHD turbulence with external forcing and mean magnetic field. This is arguably because the mean magnetic field is not exactly *B_y_*, and the perpendicular component of the wave number contaminates *k_y_*.

**Fig. 4.**
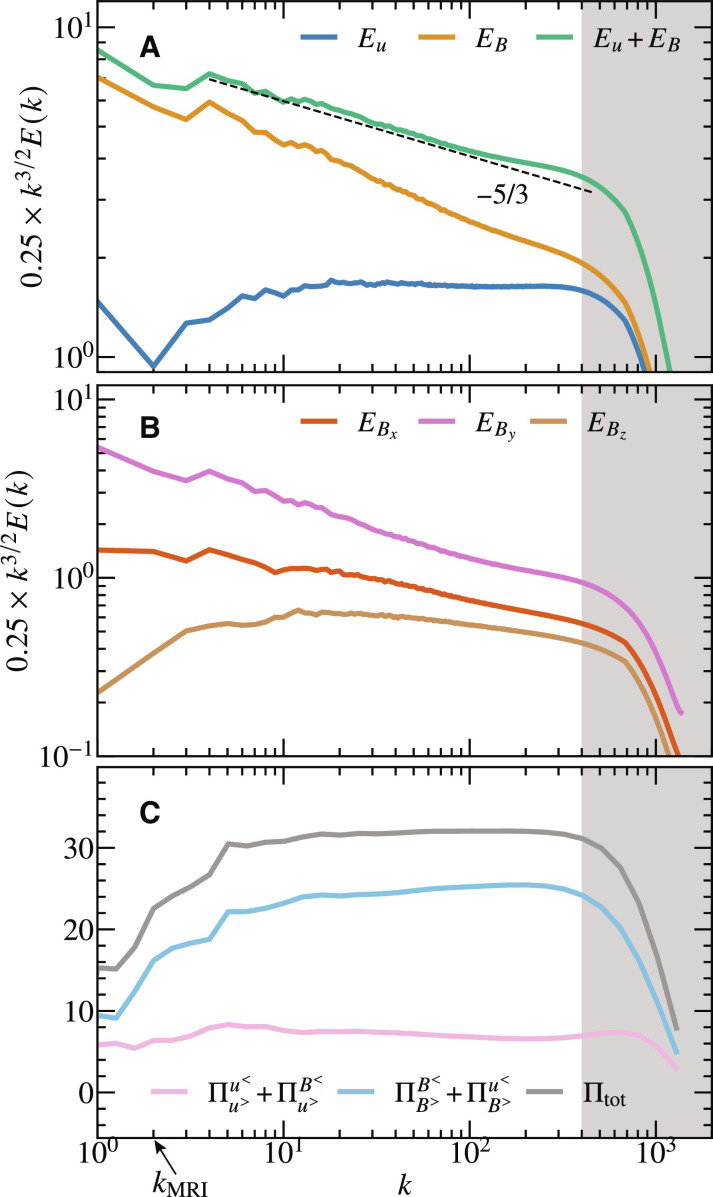
Spectra of magnetorotational turbulence. (**A**) Kinetic, magnetic, and total energy spectra compensated by *k*^3/2^, where *k* is the wave number. (**B**) Spectra of *x*, *y*, and *z* components of magnetic energy. (**C**) Cross-scale energy flux $\Pi_{g^{>}}^{f^{<}}$ denoting the transfer from the field *f* (which is the flow field when *f* = *u* and is the magnetic field when *f* = *B*) with the wave number smaller than *k* to the field *g* with the wave number larger than *k*. The gray shaded area indicates to the dissipation range. The arrow indicates the wave number *k*_MRI_ = 2 which denotes the wave number of the fastest growing MRI modes.

Figure 4A shows the omnidirectional spectra of kinetic, magnetic, and total energy. The total energy spectrum is well fitted by a power law of *k*^−5/3^ when *k* ≲ 100, where *k* is the wave number, and the spectral slope gets slightly shallower when *k* ≳ 100. While the −5/3 spectrum was captured in the previously highest-resolution simulation (*15*), the shallowing of the slope is reported here. We found that the shallowing occurs only in the magnetic field, while the kinetic energy spectrum is almost completely *k*^−3/2^ throughout the wave number domain. In the previously highest-resolution simulation (*15*), the kinetic energy spectrum was slightly shallower than *k*^−3/2^. This is likely due to insufficient numerical resolution because the kinetic energy spectrum becomes shallower than *k*^−3/2^ in our low-resolution simulation to be shown below.

The shallowing of the magnetic energy spectrum is not unexpected for the following reason. The simulation of MHD turbulence with external forcing indicates that both magnetic and kinetic energy spectra converge to *k*^−3/2^ with the same magnitude (*30*). Meanwhile, as the cascade proceeds, the eddy turnover time decreases, causing fluctuations to lose memory of their MRI origin and eventually align with the above prediction (*15*, *28*). At large scales, magnetic fluctuations are greater than those of kinetic energy and have a steeper spectrum, whereas the kinetic energy spectrum is already *k*^−3/2^, as shown in Fig. 4A. Thus, the magnetic energy spectrum must flatten to merge into the kinetic energy spectrum.

We further investigate the magnetic energy spectrum by plotting *x*, *y*, and *z* components in Fig. 4B. As is consistent with the previous simulation (*15*), *B_y_* is dominant on the large scale (due to amplification via the Ω effect) and diminishes quickly in the smaller scales. However, unlike the previous simulation, the *B_y_* spectrum becomes shallower at *k* ≳ 100, at which the total energy spectrum starts to become shallower than −5/3. The second dominant component on the large scale is *B_x_*, which is presumably due to MRI. Although one might doubt that the shallowing of the spectrum is the numerical roll-up due to the hyperdissipation, we do not think that this is the case because of the following two reasons. First, only the *B_y_* spectrum shows shallowing, while the other fields do not. Second, the shallowing disappears in our low–numerical resolution simulation, as we will show below. We will further investigate this shallowing of *B_y_* later in this paper. Although these two points do not rule out the possibility of numerical roll-up completely, it is now computationally impossible to validate the presence of shallowing using Laplacian dissipation.

Evidence that our simulation resolved the inertial range is provided in Fig. 4C, which plots the cross-scale energy flux through the wave number shell ∣**k**∣ = *k*. Here, the energy flux $\Pi_{g^{>}}^{f^{<}}\left(k\right)$ denotes the energy transfer from the field *f*, where *f* = *u* (or =*B*) for the flow (or magnetic) field, with the wave number smaller than *k* to the field *g* with the wave number larger than *k* (see Methods for the mathematical definition). We find that the total energy flux is fairly constant at *k* ≳ 10, which means that the cascade at *k* ≳ 10 is in the inertial range. We stress that, in the previous simulations of MRI turbulence, the cross-scale energy flux was not constant (*14*), and our simulations found the constant energy flux. Note, however, that not only the number of grid points but also the size of the simulation box and the aspect ratio of their simulation are different from ours. We also find that the amount of energy flux “from *u* and *B* to *B*” dominates that “from *u* and *B* to *u*,” which is consistent with the observation in (*14*).

**Fig. 5.**
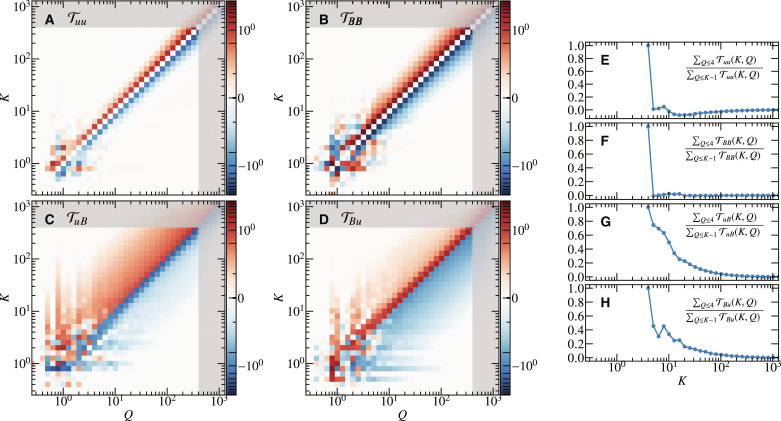
Locality of nonlinear energy transfer. (**A** to **D**) Shell-to-shell energy transfer function $T_{\text{fg}}$, representing the transfer from field *f* (the flow field for *f* = *u* and the magnetic field for *f* = *B*) with wave number *Q* to field *g* with wave number *K*. (**E** to **H**) Contribution of the energy transfer from the injection range *k* ≤ 4.

The nonlinear energy transfer is further investigated in Fig. 5. We define the transfer function $T_{\text{fg}}(Q,K)$ that denotes the energy transfer from the field *f* in the wave number shell *Q* ≤ ∣**k**∣ < *Q* + 1 to the field *g* in the wave number shell *K* ≤ ∣**k**∣ < *K* + 1 (*31*–*34*) (see Methods for the mathematical definition). We find that the dominant energy transfer among all possible combinations of fields is $T_{\text{BB}}$, which is consistent with (*14*). Noticeably, there is a transition at ∣**k**∣ ≈ 4. In ∣**k**∣ > 4, both $T_{\text{uu}}$ and $T_{\text{BB}}$ are fairly local, and the direction of the cascade is forward. On the other hand, in ∣**k**∣ < 4, none of the energy transfers are local. We also find that there is an inverse energy transfer for $T_{\text{BB}}$, suggesting that the large-scale structures of the magnetic field shown in Fig. 1A is formed not just by the Ω effect but also by the inverse cascade. Furthermore, the transition scale, ∣**k**∣ ≈ 4, coincides with the scale where the energy flux becomes constant (Fig. 4C), meaning that the energy cascade in the inertial range is local. Regarding the energy transfers between *u* and *B*, we find that they are more nonlocal than the transfer within *u* or *B* as $T_{\text{uB}}$ and $T_{\text{Bu}}$ have broad off-diagonal tails. That being said, the nonlocal transfer from the injection range (∣**k**∣ ≤ 4) peters out as the cascade proceeds. This is evident from Fig. 5 (E to H), which shows the contribution of energy transfer from the injection range defined by ∑_*Q*≤4_ T*_fg_*(*K*, *Q*)/∑_*Q* ≤ *K*−1_ T*_fg_*(*K*, *Q*). We find that the contribution of the injection range in $T_{\text{uu}}$ and $T_{\text{BB}}$ disappears immediately below the transition scale ∣**k**∣ = 4, which is obvious since these transfers are fairly local, as mentioned above. For $T_{\text{uB}}$ and $T_{\text{Bu}}$, the contribution of the injection range survives down to the relatively smaller scale, but the contribution becomes less than 10% at ∣**k**∣ = 50 for $T_{\text{uB}}$ and at ∣**k**∣ = 40 for $T_{\text{Bu}}$. This is markedly different from (*14*), which showed that the box-scale to grid-scale transfer of $T_{\text{uB}}$ was substantial. In short, the two facts that the cross-scale energy flux is constant and that the nonlocal energy transfer from the injection range peters out manifest that the cascade in our simulation is in the inertial range. This is one of the two main results of this paper.

### Partition between slow magnetosonic and Alfvénic fluctuations

**Fig. 6.**
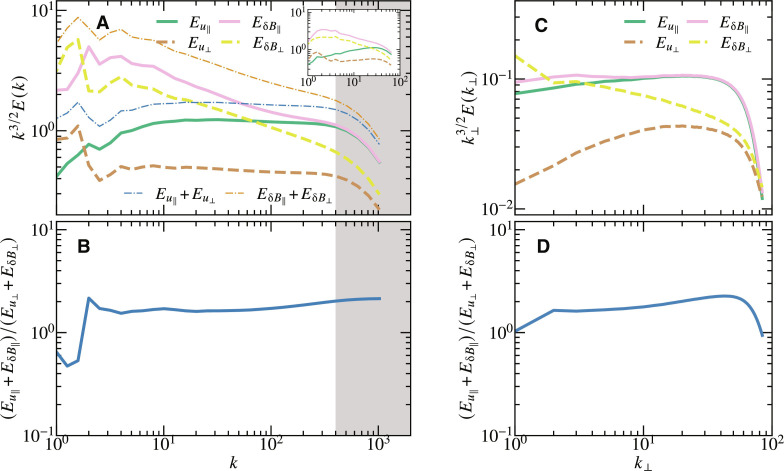
Decomposition of flow and magnetic fields into Alfvén and slow magnetosonic fluctuations. (**A** and **C**) Spectra of Alfvén and slow magnetosonic fluctuations, respectively. The inset in (A) shows the spectra obtained by the low-resolution simulation with *N_x_* × *N_y_* × *N_z_* = 512 × 512 × 256. (**B** and **D**) Ratio of slow magnetosonic to Alfvénic fluctuations. (A) and (B) are obtained from the MHD simulation presented in this paper, while (C) and (D) are obtained from the reduced MHD simulation (*28*).

The other main result of this paper, namely, the partition between slow magnetosonic and Alfvénic fluctuations in the magnetorotational turbulence, is shown in Fig. 6. In incompressible MHD, the flow and magnetic field of the Alfvén waves are described by $u_{⊥}{\hat{k}}_{∥}\times {\hat{k}}_{⊥}$ and $\delta B_{⊥}{\hat{k}}_{∥}\times {\hat{k}}_{⊥}$ while those of the slow magnetosonic waves are described by $u_{∥}\left[{\hat{k}}_{∥}-\left(k_{∥}/k_{⊥}\right){\hat{k}}_{⊥}\right]$ and $\delta B_{∥}\left[{\hat{k}}_{∥}-\left(k_{∥}/k_{⊥}\right){\hat{k}}_{⊥}\right]$ , respectively, where the hat symbol denotes a unit vector, and the eigenfunction of the slow magnetosonic waves is used. As we found in Figs. 1 and 3, the wave number is strongly anisotropic, i.e., *k*_∥_/*k*_⊥_ ≪ 1. This allows the slow magnetosonic waves to be represented simply by *u*_∥_ and δ*B*_∥_ (fig. S3 confirms that neglecting the terms proportional to *k*_∥_/*k*_⊥_ does not change the spectra). Thus, we can decompose the total magnetic and kinetic energy into those of Alfvénic and slow magnetosonic fluctuations via projection of **u** and **B** onto the mean magnetic field. However, the global mean magnetic field does not always serve as a mean field for small-scale fluctuations, and thus, we use the method developed by Cho and Lazarian (*35*) to decompose the fluctuation and the local mean magnetic field **B**_0_(**r**). For a given wave number *k*, the local mean magnetic field **B**_0_(**r**) is obtained by filtering the Fourier modes of **B** with the wave number greater than *k*/2, and the fluctuations are obtained by filtering out the Fourier modes of **u** and **B** with the wave number smaller than *k*/2 or greater than 2*k*. Then, we decompose the fluctuations of **u** and **B** into parallel (*u*_∥_ and δ*B*_∥_) and perpendicular (*u*_⊥_ and δ*B*_⊥_) components to **B**_0_. Figure 6A shows the spectra of the decomposed fields. First, we confirm the validity of our decomposition by comparing the sum of *u*_∥_ and *u*_⊥_ with the total *u* in Fig. 1A and the sum of δ*B*_∥_ and δ*B*_⊥_ with the total *B* in Fig. 1A; the spectra of the total fields are almost perfectly recovered from the sum of the decomposed fields. In terms of magnetic fluctuations, one finds that only the spectrum of slow magnetosonic fluctuations exhibits flattening at *k* ≳ 100, while that of Alfvénic fluctuations has nearly the same spectral index throughout the inertial range. In contrast, the kinetic energy spectra of both Alfvén and slow magnetosonic fluctuations are almost perfectly *k*^−3/2^. Therefore, the flattening of the spectrum seen in Fig. 1A is solely due to the magnetic component of slow magnetosonic fluctuations. The inset of Fig. 6A shows the same spectra obtained from the simulation with lower resolution *N_x_* × *N_y_* × *N_z_* = 512 × 512 × 256, which manifests that the flattening of the magnetic energy of the slow magnetosonic waves is absent. Thus, the flattening can be seen only with ultrahigh resolution. It can be seen that due to this flattening, the magnetic energy and kinetic energy of slow magnetosonic fluctuations tend to be equipartitioned, and both spectra approach *k*^−3/2^. On the other hand, the kinetic and magnetic energy spectra of Alfvénic fluctuations do not converge in our simulation, and a further higher resolution is required to determine the converged spectral slope. Figure 6B shows the ratio of the energy of slow magnetosonic fluctuations to that of Alfvénic fluctuations, manifesting that slow magnetosonic fluctuations have approximately twice stronger energy than the Alfvénic ones. One also finds that the ratio is almost constant throughout the wave number domain, suggesting that the coupling between the Alfvénic and slow magnetosonic fluctuations in MRI turbulence is weak. This is consistent with the recent report on the shearing box simulation which found that the ratio of energy injection between Alfvénic and slow magnetosonic fluctuations almost equals to the ratio of dissipation which we computed using the reduced MHD (*36*).

Last, we compare our results with the simulation of magnetorotational turbulence solved by the reduced MHD with a near-azimuthal mean magnetic field (*28*). Figure 6 (C and D) is reproduced from (*28*) and shows qualitative consistency with our results in Fig. 6 (A and B). Specifically, Fig. 6C displays the spectra of the slow magnetosonic and Alfvénic fluctuations, which are remarkably similar to the spectra at *k* ≳ 100 in Fig. 6A. Figure 6D shows the ratio between the two fluctuations, with a value of ≈2, exactly matching the result in Fig. 6B. Thus, we conclude that the reduced MHD with a near-azimuthal mean magnetic field effectively captures the features of the inertial range of magnetorotational turbulence.

## DISCUSSION

The validity of reduced MHD in solving the inertial range of magnetorotational turbulence is now supported by the two findings of this study, namely, (i) that the spatial structures of the small-scale fluctuations in our magnetorotational turbulence are elongated along the large-scale magnetic field which is azimuthally elongated and (ii) that their spectral shapes remarkably resemble those obtained by reduced MHD. Although the results of our MHD simulation are formally applicable when β, thermal-to-magnetic pressure ratio, is infinitely large as we solved incompressible MHD, the simulations of reduced MHD showed that the spectral shape and the energy partition between the slow magnetosonic and Alfvénic fluctuations (viz., by the factor of two) do not depend on β. Thus, we think that the results shown in this paper are also valid in the smaller β regime. Note that the recent study shows that the ratio of energy injection between the slow magnetosonic and Alfvénic fluctuations does not depend on β (*36*), which supports our claim. Apart from β, our results can depend on the value of Pm and on the presence or absence of net magnetic flux. While we have explored only the case with Pm = 1, with finite vertical net flux and without azimuthal net flux, numerous simulations have investigated other cases [e.g., see (*37*) for the latest study]. However, these previous parameter scans mostly focus on the dependence of the value of α and saturation amplitude. Therefore, it would be very interesting to investigate whether the results of this study (e.g., the partition between slow magnetosonic and Alfvénic fluctuations) change under different settings.

The energy partition between the slow magnetosonic and Alfvénic fluctuations is important for inferring the ion versus electron heating in hot accretion flows, such as at M87 and Sgr A*. The ion-to-electron heating ratio is a key parameter for theoretical understanding of radio observations by the Event Horizon Telescope (*38*). In collisionless magnetorotational turbulence, the half of the energy flux injected via MRI is supposed to be viscously dissipated due to the pressure anisotropy (*39*, *40*), and the remaining half turns into ion and electron heating at the microscopic scales smaller than the ion Larmor radius. Our previous study of microscopic turbulence using hybrid gyrokinetics showed that, regarding the heating at the microscopic scales, ions are heated more efficiently than electrons when slow-mode-like compressive fluctuations dominate the Alfvénic ones (*10*). Thus, the fact that the slow magnetosonic fluctuations have twice as large energy as the Alfvénic ones indicates that at least half of the energy cascaded down to the ion Larmor scale dissipates into ion heating [see also (*28*) for some caveats of this conclusion]. Recently, the one-dimensional energy transport model of hot accretion disks showed that the preferential ion heating due to the slow-mode-like compressive fluctuations can substantially influence the global temperature distribution (*41*). We should note that this discussion assumes that MRI turbulence is active in the accretion flows around M87 and Sgr A* (*42*, *43*). However, the theoretical examinations of the Event Horizon Telescope data prefer magnetically arrested disk (MAD) regime, in which MRI is supposed to be strongly (but not completely) suppressed due to strong magnetic fields. That being said, the contribution of MRI-driven turbulence to the radiation profile in a MAD state is still being debated. Furthermore, the accumulation and amplification of the poloidal magnetic field due to accretion caused by MRI are crucial for achieving the MAD state (*44*, *45*). There is also a study that suggests MRI is not even suppressed in MADs, contrary to previous claims (*46*).

MRI turbulence can accelerate high-energy nonthermal particles via magnetic reconnection (*29*, *47*–*49*), and these higher-energy particles are further accelerated via stochastic acceleration through wave-particle interactions (*50*). If nonthermal protons are accelerated to higher energies, then these protons can produce cosmic neutrinos seen by IceCube experiments (*27*). Previous studies on stochastic acceleration using MHD and test particle simulations exhibit particle acceleration with a hard-sphere–type diffusion coefficient in momentum space (*5*, *6*, *24*). However, these MHD simulations lack sufficient spacial resolution, underestimating the acceleration efficiency at lower energies. Our highest-resolution MRI simulation resolves the inertial range of MRI turbulence. This allows us to evaluate the particle acceleration efficiency at much lower energies down to dissipation scale. This would shed light on modeling particle acceleration inside accretion flows.

Last, when one aims to explore the inertial range of magnetorotational turbulence, extremely expensive full MHD simulations (like we did in this study) are not necessary, and simulations of reduced MHD with a small amount of computational cost would be enough. The adequacy of reduced MHD opens up the possibility of exploring magnetorotational turbulence in the collisionless regime where the MHD approximation formally breaks down. There have been a number of numerical studies of collisionless magnetorotational turbulence [e.g., (*29*, *39*, *40*, *47*–*49*, *51*–*55*)], but presumably, the inertial range was not well resolved because the models used in these studies are much more complicated and numerically harder to solve than MHD. However, this study suggests that it is possible to reach the inertial range of collisionless magnetorotational turbulence using the reduced kinetic MHD (*8*) in a rotating frame, whose collisional limit is the reduced MHD which we used in (*28*).

## METHODS

### Governing equations

We consider a local Cartesian coordinate that corotates with the accretion disk at a radial distance *r* = *r*_0_ from the center. The coordinate labels (*x*, *y*, *z*) denote the radial, azimuthal, and vertical directions, respectively. We solve the incompressible MHD equations in this coordinate system

$$\frac{\partial u}{\partial t}+\left(u_{0}+u\right)\cdot \nabla u=-\nabla P+B\cdot \nabla B-2\Omega \hat{z}\times u-u\cdot \nabla u_{0}$$ (1)

$$\frac{\partial B}{\partial t}+\left(u_{0}+u\right)\cdot \nabla B=B\cdot \nabla \left(u_{0}+u\right)$$ (2)

∇·u=0,∇·B=0 (3)

where **u** is the flow velocity, **B** is the magnetic field, *P* is the total pressure, Ω is the local angular velocity of the disk, *q* = −(dlnΩ/dln*r*)_*r*=*r*_0__ is the shear rate, and $u_{0}=-q\Omega x\hat{y}$ is the background flow. Because of the incompressible condition, the density ρ is spatiotemporally constant. Although MRI turbulence can excite substantial acoustic wave power in accretion flows, the MRI turbulence itself is highly incompressible. The particle-in-cell simulation demonstrated that the incompressible approximation is valid in collisionless magnetorotational turbulence in a shearing box (*56*). We assume that the rotation of the disk is Keplerian, i.e., *q* = 3/2. The boundary condition is set to periodic in *y* and *z* and to shearing periodic in *x* (*11*).

### Numerical setup

We numerically solve Eqs. 1 to 3 via pseudo-spectral code Calliope (*57*, *58*). To adopt the pseudospectral method, we enforce the triply periodic boundary conditions by transforming to the shearing coordinate, *y* ↦ *y* − *q*Ω*tx*. This transformation makes the radial wave number time dependent, *k_x_*(*t*) = *k_x_* + *q*Ω*tk_y_*, where *k_x_* and *k_x_*(*t*) are the radial wave number in the shearing frame and the laboratory frame, respectively. To avoid *k_x_* from ever growing, we adopt the remapping method where the fields are mapped to the original nonshearing coordinate every *T* = *L_y_*/(*q*Ω*L_x_*) (*59*–*60*).

We set the initial magnetic field as the sum of the uniform vertical field $B_{0}\hat{z}$ and the random fluctuations with amplitude much smaller than *B*_0_. The size and aspect ratio of the computational domain are set to *L_x_* × *L_y_* × *L_z_* = 4λ × 8λ × 2λ, where λ = 2π*v*_A_/Ω approximately equals the wavelength of the fastest-growing modes of MRI (*61*) and $v_{A}=B_{0}/\sqrt{4\pi \rho }$ is the Alfvén speed given by the initial magnetic field.

The computational domain was discretized into *N_x_* × *N_y_* × *N_z_* = *N* × *N* × *N*/2 grid points. We set *N* at 256 initially and gradually increased it to 8192 after nonlinear saturation. Each time *N* was increased, we continued the simulation until the spectral shapes near the dissipation scale did not change before *N* was increased again. When *N* was increased to 8192, the simulation was continued for a duration exceeding 100 Ω^−1^ from the initial time. Thus, the MRI turbulence near the injection scale was sufficiently developed before we start the highest-resolution run.

As the numerical resolution increases, the simulation timestep must be decreased to satisfy the Courant-Friedrichs-Lewy condition. We only computed for ≃0.8 Ω^−1^ after *N* increased from 4096 to 8192, and it was impossible to execute the highest-resolution run over multiple eddy turnover time although we exhausted ≃112 million CPU hours. However, we found that the spectral shapes at small scales (*k* > 10) do not depend on time, as we can see in fig. S2, which shows the time history of spectra during the last 0.6 Ω^−1^ after the resolution increased (i.e., at *t* = 0 in fig. S3, approximately 0.2 Ω^−1^ passed after *N* was increased).

To broaden the inertial range, we used fourth-order hyperviscosity ν_h_∇^4^**u** and hyperresistivity η_h_∇^4^**B** to terminate the cascade. Magnetic Prandtl number Pm = ν_h_/η_h_ was set to unity. We found that when the order of the hyperdissipation was eight, there appeared an unphysical roll up in the kinetic energy spectrum.

### Shell-to-shell energy transfer function and cross-scale energy flux

We first introduce the filtering in the wave number shell ∣**k**∣ = *K*

$$u_{K}\left(x\right)=\sum_{K\leq |k|<K+1}‍{\hat{u}}_{k}e^{ik\cdot x},\;B_{K}\left(x\right)=\sum_{K\leq |k|<K+1}‍{\hat{B}}_{k}e^{ik\cdot x}$$ (4)

where ${\hat{u}}_{k}$ and ${\hat{B}}_{k}$ are the Fourier coefficients of **u** and **B**, respectively. Then, the energy transfer between the field *f* in the shell *Q* ≤ ∣**k**∣ < *Q* + 1 to the field *g* in the shell *K* ≤ ∣**k**∣ < *K* + 1, denoted by $T_{\text{fg}}(Q,K)$, is calculated as (*31*–*34*)

$$T_{\mathrm{uu}}\left(Q,K\right)=-\int ‍d^{3}r\left[u_{K}\cdot \left(u\cdot \nabla u_{Q}\right)\right]$$ (5)

$$T_{\mathrm{BB}}\left(Q,K\right)=-\int ‍d^{3}r\left[B_{K}\cdot \left(u\cdot \nabla B_{Q}\right)\right]$$ (6)

$$T_{\mathrm{uB}}\left(Q,K\right)=\int ‍d^{3}r\left[B_{K}\cdot \left(B\cdot \nabla u_{Q}\right)\right]$$ (7)

$$T_{\mathrm{Bu}}\left(Q,K\right)=\int ‍d^{3}r\left[u_{K}\cdot \left(B\cdot \nabla B_{Q}\right)\right]$$ (8)

Integrating by parts, we obtain the identity $T_{\text{fg}}(Q,K)=-T_{\text{fg}}(K,Q)$. The cross-scale energy flux across the wave number shell ∣**k**∣ = *K* is defined as the transfer from all scales larger than *K* to all scales smaller than *K*. It is given in (*34*)

$$\Pi_{g^{>}}^{f^{<}}\left(k\right)=\sum_{Q<k}‍\sum_{K>k}‍T_{\mathrm{fg}}\left(Q,K\right)$$ (9)
