# Supplementary material for: Inertial range of magnetorotational turbulence
Source: Sci Adv. 2024 Aug 28;10(35):eadp4965. doi: 10.1126/sciadv.adp4965 (PMC11352918; doi:10.1126/sciadv.adp4965)
Supplement: Supplementary file 1 — Figs. S1 to S3 [file sciadv.adp4965_sm.pdf]

Science Advances
AAAS

Supplementary Materials for

Inertial range of magnetorotational turbulence

Yohei Kawazura and Shigeo S. Kimura

Corresponding author: Yohei Kawazura, kawazura@a.utsunomiya-u.ac.jp

Sci. Adv. **10**, eadp4965 (2024)
DOI: 10.1126/sciadv.adp4965

This PDF file includes:

Figs. S1 to S3

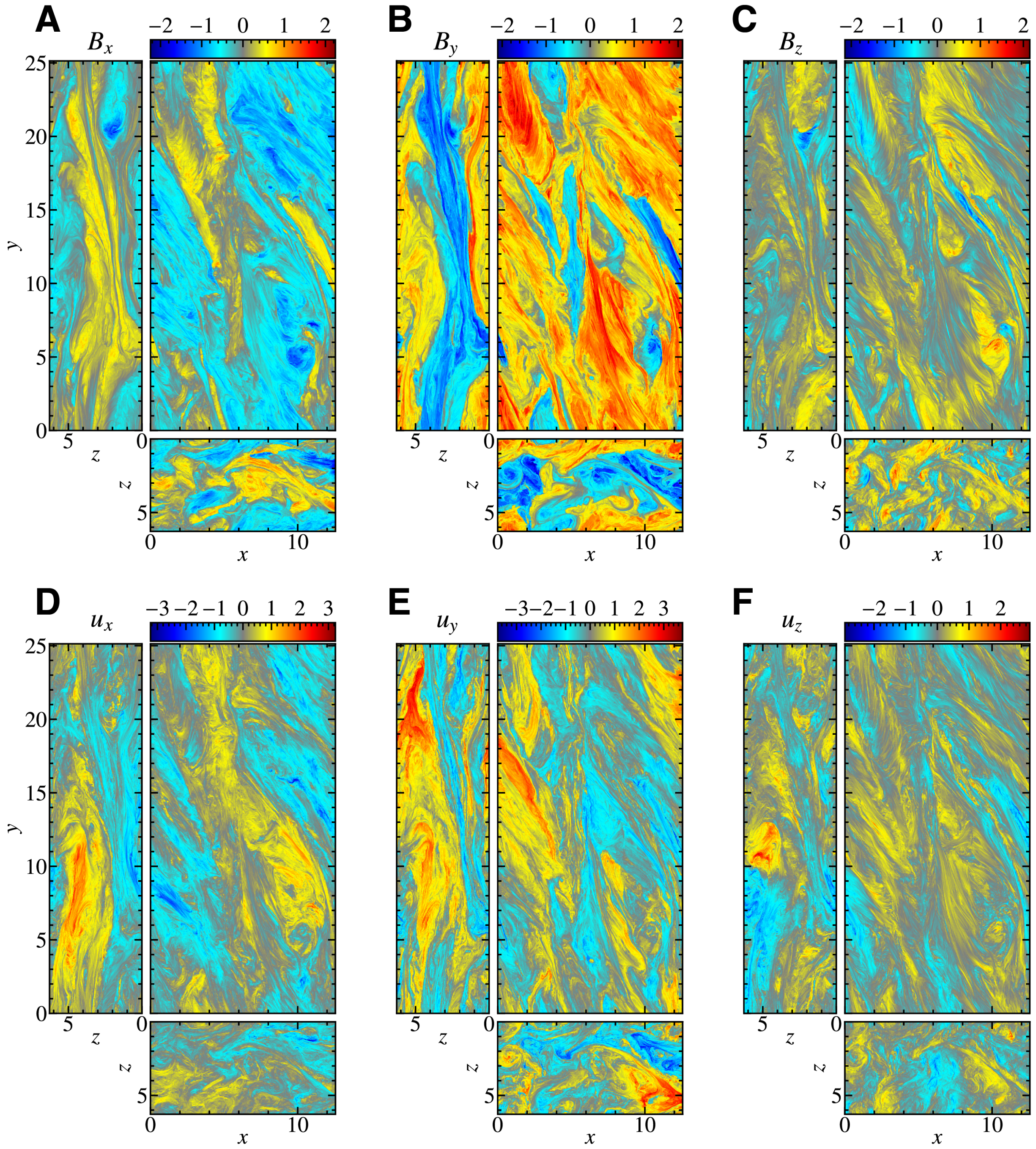

Figure S1: **The spatial distribution of the vector components of magnetic and flow fields.** **(A)**, **(B)**, and **(C)** for the magnetic fields B_x, B_y, and B_z and **(D)**, **(E)**, and **(F)** for the flow fields u_x, u_y, and u_z. The snapshot was taken at the same time as in Fig. 1. One finds that only B_y has intense large-scale structures, which are supposed to be created by the Ω effect and inverse cascade, as mentioned in the main text.

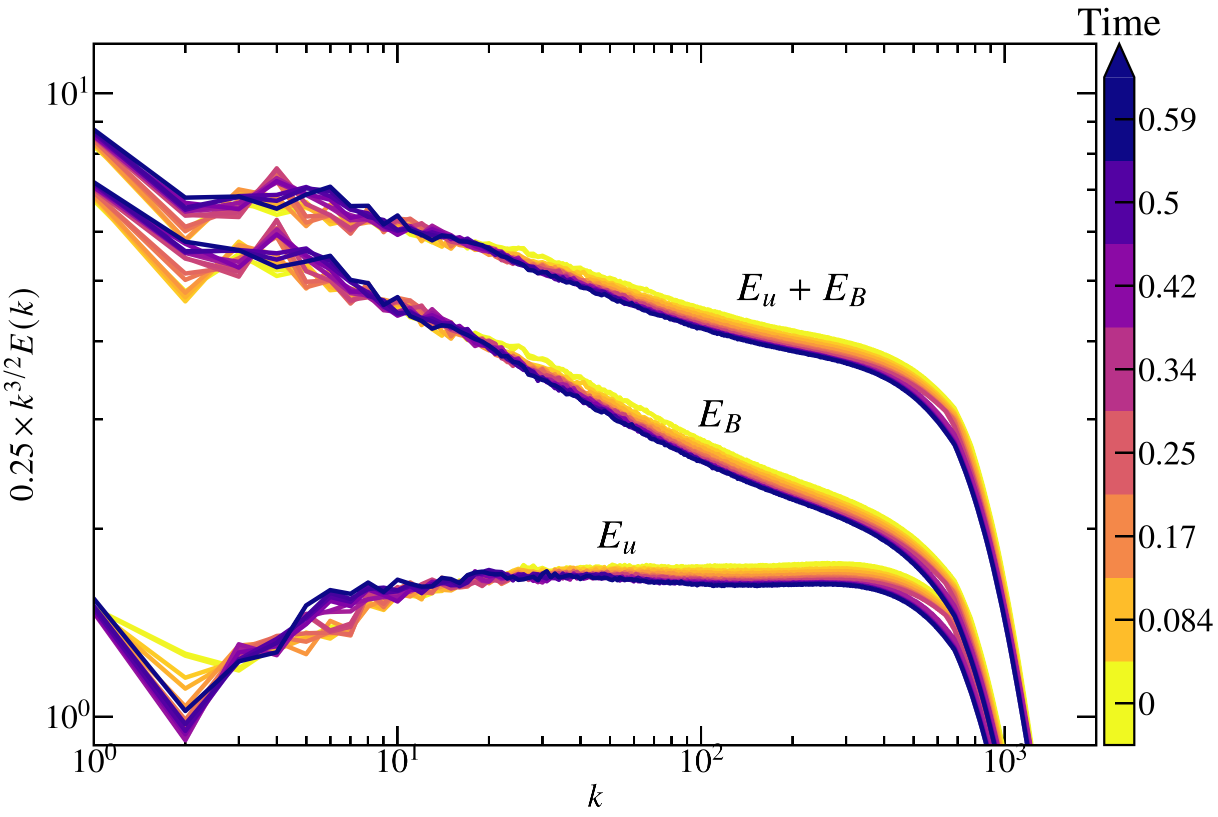

Figure S2: **Time evolution of spectra.** The evolution is displayed from $t = 0$ to $0.6\Omega^{-1}$ where $t = 0$ corresponds to the time when $0.2\Omega^{-1}$ passed after the resolution was increased to $N = 8192$.

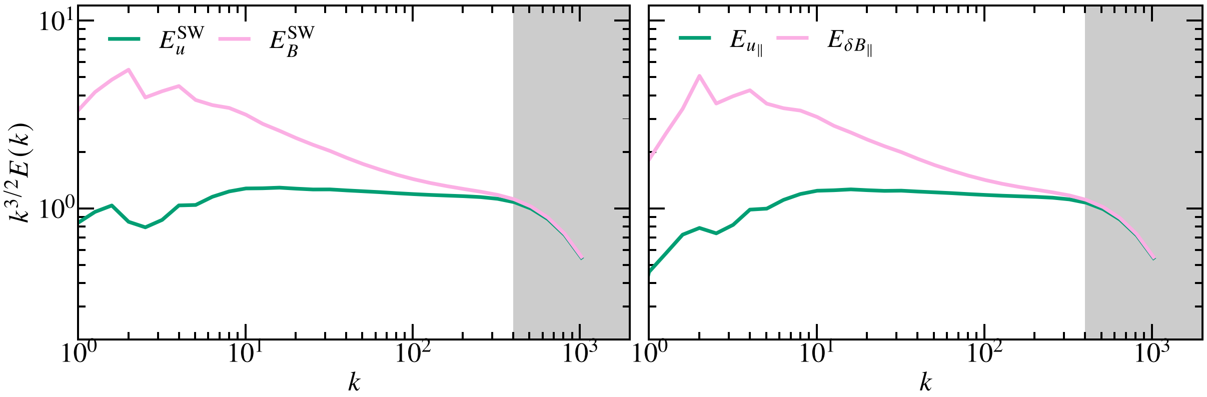

Figure S3: **Comparison between the spectra of slow-magnetosonic fluctuations and parallel fluctuations.** (Left) The spectrum of magnetic and kinetic energy of slow-magnetosonic waves $E_u^{\rm SW} = (1+k_\parallel^2/k_\perp^2)u_\parallel^2/2$ and $E_B^{\rm SW} = (1+k_\parallel^2/k_\perp^2)\delta B_\parallel^2/2$, respectively, and (Right) the spectrum of $u_\parallel^2/2$ and $\delta B_\parallel^2/2$ (the same as Fig. 6A). The only difference is at low-k region were the anisotropy $k_\parallel/k_\perp \ll 1$ is not yet developed.
